# Inverse agonism of retinoic acid receptors directs epiblast cells into the paraxial mesoderm lineage

**DOI:** 10.1016/j.scr.2018.05.016

**Published:** 2018-05-22

**Authors:** Ryan P. Russell, Yu Fu, Yaling Liu, Peter Maye

**Affiliations:** Department of Reconstructive Sciences, School of Dental Medicine, University of Connecticut Health Center, United States

**Keywords:** Embryonic stem cell, Paraxial mesoderm, Axial skeleton, Epiblast, Retinoic acid

## Abstract

We have investigated the differentiation of paraxial mesoderm from mouse embryonic stem cells utilizing a *Tbx6-EYFP/Brachyury* (*T*)-*Ckerry* dual reporter system. Differentiation from the mouse ESC state directly into mesoderm via Wnt pathway activation was low, but augmented by treatment with AGN193109, a pan-retinoic acid receptor inverse agonist. After five days of differentiation, T^+^ cells increased from 12.2% to 18.8%, Tbx6^+^ cells increased from 5.8% to 12.7%, and T^+^/Tbx6^+^ cells increased from 2.4% to 14.1%. The synergism of AGN193109 with Wnt3a/CHIR99021 was further substantiated by the increased expression of paraxial mesoderm gene markers *Tbx6, Msgnl, Meoxl,* and *Hoxbl.* Separate to inverse agonist treatment, when mouse ESCs were indirectly differentiated into mesoderm via a transient epiblast step the efficiency of paraxial mesoderm formation markedly increased. Tbx6^+^ cells represented 65–75% of the total cell population after just 3 days of differentiation and the expression of paraxial mesoderm marker genes *Tbx6* and *Msgn* increased over 100-fold and 300-fold, respectively. Further evaluation of AGN193109 treatment on the indirect differentiation protocol suggested that RARs have two distinct roles. First, AGN193109 treatment at the epiblast step and mesoderm step promoted paraxial mesoderm formation over other mesoderm and endoderm lineage types. Second, continued treatment during mesoderm formation revealed its ability to repress the maturation of presomitic mesoderm into somitic paraxial mesoderm. Thus, the continuous treatment of AGN193109 during epiblast and mesoderm differentiation steps yielded a culture where —90% of the cells were Tbx6^+^. The surprisingly early effect of inverse agonist treatment at the epiblast step of differentiation led us to further examine the effect of AGN193109 treatment during an extended epiblast differentiation protocol. Interestingly, while inverse agonist treatment had no impact on the conversion of ESCs into epiblast cells based on the expression of *Rexl, Fgf5,* and pluripotency marker genes *Oct4, Nanog,* and *Sox2,* after three days of differentiation in the presence of AGN193109 caudal epiblast and early paraxial mesoderm marker genes, T, *Cyp26al, Fgf8, Tbx6* and *Msgn* were all highly up-regulated. Collectively, our studies reveal an earlier than appreciated role for RARs in epiblast cells and the modulation of their function via inverse agonist treatment can promote their differentiation into the paraxial mesoderm lineage.

## 1. Introduction

The differentiation of pluripotent stem cells can be used as a valuable approach to study development, disease and obtain therapeutic progenitor cells. Our interest in skeletal biology has motivated us to learn how to differentiate embryonic stem cells (ESCs) into paraxial mesoderm. The paraxial mesoderm is a highly desirable cell population to generate because it is the precursor that gives rise to all of the cartilage, bone, skeletal muscle, and tendons that comprise the axial skeleton.

An increasing body of work has provided crucial insight into the mechanisms that instruct paraxial mesoderm formation. Two transcription factors, *T-box 6* (*Tbx6*) and *Mesogenin* (*Msgn*) are pivotal regulators in the transition of caudal epiblast cells into unsegmented paraxial mesoderm, which is also known as presomitic mesoderm. Targeted loss of *Tbx6* in mice resulted in the formation of ectopic neural tubes at the expense of paraxial mesoderm formation (Chapman and Papaioannou, 1998). Tbx6 represses *Sox2* expression to direct caudal epiblast cells into the paraxial mesoderm lineage (Nowotschin et al., 2012; Takemoto et al., 2011). Msgn orchestrates the differentiation and migration of progenitor cells exiting the tailbud which form the presomitic mesoderm, and is therefore critical for balancing paraxial mesoderm specification and maintenance of the caudal progenitor population (Chalamalasetty et al., 2014; Fior et al., 2012). Together, Tbx6 and Msgn control the differentiation and maturation of paraxial mesoderm required for proper embryo elongation.

Wnt and FGF signaling pathways have essential roles in the lineage specification and outgrowth of axial progenitor cells. Targeted loss of *Wnt3a* (Yoshikawa et al., 1997) and *Lef1/Tcf1* double mutants (Galceran et al., 1999) resulted in a phenotype similar to loss of *Tbx6* in that broader contribution of epiblast cells into the neural ectoderm fate at the cost of forming paraxial mesoderm. With that in mind, there is an overwhelming amount of genetic and biochemical data showing that canonical Wnt signaling plays a major role in mesoderm formation. Gene targeting studies in mice have shown how several members of the Wnt pathway are required for mesoderm formation including, *Wnt3, β- Catenin,* and *LRP5/LRP6* double mutants (Huelsken et al., 2000; Kelly, 2004; Liu et al., 1999). During ESC differentiation, Wnt3a and GSK3β antagonists are commonly used to induce mesoderm formation with higher levels of canonical Wnt activity promoting the formation of paraxial mesoderm (Craft et al., 2013; Gadue et al., 2006; Lindsley, 2006; Tanaka et al., 2009; Umeda et al., 2012). FGF signaling is indispensable during embryogenesis, as knockouts of *Fgf4, Fgf8, Fgfr1, or Fgfr2* are all early embryonic lethal (Arman et al., 1998; Deng et al., 1994; Feldman et al., 1995; Sun et al., 1999), and dominant negative mutants display disruption of gastrulation cell movements, lack of mesoderm specification, and severe caudal truncation (Amaya et al., 1991; Fletcher and Harland, 2008). FGF signaling in epiblast cells promotes migration through the primitive streak to initiate mesoderm formation and patterning, and a high FGF concentration persists in the tailbud to maintain the pluripotency and proliferation of cells contributing to the posterior paraxial mesoderm (Boulet and Capecchi, 2012; Burdsal et al., 1998; Ciruna and Rossant, 2001). Subsequent to presomitic mesoderm formation, a cyclical process of maturation occurs that involves periodic segmentation to form paired somites. Two important transcription factors *Mesp2* and *Ripply2* are critical in forming the segmental border at the anterior-most region of the presomitic mesoderm thereby controlling the size and organization of each somite (Takahashi et al., 2010; Zhao et al., 2015). Functioning as negative regulators, *Mesp2* and *Ripply2* establish the anterior boundary of Tbx6, and therefore mark the extent of the presomitic mesoderm domain. A negative feedback loop exists in the PSM wherein Tbx6, along with Notch signaling, induces *Mesp2* expression which in turn induces *Ripply2* expression (Sasaki et al., 2011; Yasuhiko et al., 2008). Mesp2 and Ripply2 cause Tbx6 degradation, while Ripply2 also downregulates *Mesp2* expression to complete somite patterning (Moreno et al., 2008; Wanglar et al., 2014).

The maturation of paraxial mesoderm is also highly regulated by Retinoic Acid (RA) signaling. At the caudal end of the developing embryo, *Cyp26a1,* a gene that encodes for an enzyme which breaks down retinoic acid, is expressed thereby specifying a region absent of RA signaling (Fujii, 1997). Targeted loss of *Cyp26a1* results in severe caudal truncation, which mimics defects caused by teratogenic levels of RA (Abu-Abed et al., 2001; Sakai et al., 2001). Examination of *Wnt3a* and *Fgf8* expression in *Cyp26a1* mutants, genes normally expressed at the caudal end and important for driving axial outgrowth were down- regulated. However, as the anterior most region of presomitic mesoderm distances itself from the caudal end where *Cyp26a1* is expressed, levels of RA signaling increase in conjunction with the formation of somites and *Meox1* expression (Haselbeck et al., 1999; Mankoo et al., 2003). *Aldh1a2,* a gene that encodes for an enzyme responsible for RA synthesis is highly expressed in the somites and is necessary for proper somite development (Rhinn and Dolle, 2012). Interestingly, of the three Retinoic Acid Receptors (RARα,β,γ), RARγ and RARβ are expressed in distinct zones that correspond with the caudal tail and trunk region, zones retaining low and high retinoic acid signaling, respectively.

In this study, we have undertaken efforts to direct mouse ESCs into paraxial mesoderm. Our work has led us to become very interested in the role of RARs in this process. Here we present evidence that inverse agonism of RARs via treatment of AGN193109, a pan-RAR inverse agonist in epiblast cells and nascent mesoderm promotes their differentiation into paraxial mesoderm.

## 2. Materials and methods

### 2.1. Cell culture

Mouse ESCs were maintained on 0.1% gelatin coated tissue culture dishes (Thermo Scientific) and grown in serum-free maintenance media containing a 1:1 mixture of DMEM/F12 and Neurobasal medium (Life Technologies), N2 and B27 supplements, 0.05% BSA, 100U/ml penicillin, 100μg/ml streptomycin, 1.5 × 10^−4^ M monothioglycerol, 3μΜ CHIR99021 (Stemgent), 1μΜ PD0325901 (Cayman Chemical), and 10 ng/ml LIF (Millipore) (Nagy et al., 2003). For differentiation, mouse ESCs were seeded on tissue culture dishes coated with Geltrex (Gibco) and grown for a minimum of 24 h in maintenance media. For differentiation, cells were grown in a 3:1 mixture of IMDM and Ham’s F12 (Life Technologies) N2, B27 without vitamin A, 0.05% BSA, 100U/ml penicillin, 100μg/ml streptomycin, 1.5 × 10^−4^ M monothioglycerol, and 0.5 mM ascorbic acid. For mesoderm differentiation, 50 ng/ml Wnt3a (PeproTech), 3uM CHIR99021 (Stemgent), 1μΜ AGN193109 (Santa Cruz Biotechnology), 10 ng/ml FGF2 and 100 ng/ml Noggin (PeproTech) were added in different combinations. Epiblast induction was carried out in differentiation media containing 10 ng/ml FGF2 and 10 ng/ml Activin A (R&D Systems), with or without 1μΜ AGN193109 for the indicated durations. *Note:* While initial experiments used both Wnt3a and CHIR99021, it was determined that the potency of this combination was negligible to that of CHIR99021 alone. Therefore, subsequent experiments utilized CHIR99021 without the addition of Wnt3a.

### 2.2. Generation of fluorescent reporter ESC lines

For the creation of Tbx6-EYFP/T-Cherry dual reporter mouse ESCs, a BAC clone CTD-2379F21 (Children’s Hospital Oakland Research Institute) containing the *Brachyury* gene was engineered with a Cherry fluorescent reporter gene using bacterial recombination strategies as previously described(Gong et al., 2002). In brief, a homology arm was PCR amplified from the BAC clone using *Pfx* DNA polymerase (Life Technologies) with primers 5’-CTCTGCGGCCGCACTGAATTTCGGTCC CCAGAGA-3’ (sense), 5’-CTCTGGATCCGAAGCCCAGACTCGCTACC TGA-3’ (antisense). The DNA fragment was cloned into the Not1- BamHl site of pLD53.SC2-Cherry and Rec A was used to integrate the reporter into the BAC clone. The BAC clone was then retrofitted with puromycin resistance through Cre/LoxP recombination by co-electro- porating pCTP, which expresses Cre recombinase and pUni, which contains an EF1α-puromycin resistance gene and a LoxP site into CTD- 2379F21 competent bacteria. Purified BAC was transfected into Tbx6- H2B-EYFP ESCs (generously provided by Sonja Nowotschin and Katerina Hadjantonakis (van den Brink et al., 2014)) using Lipofectamine 2000 (Life Technologies) and clones were enriched by puromycin selection and screened for the transgene by PCR genotyping using 5’-CTCTGCGGCCGCACTGAATTTCGGTCCCCAGAGA-3’ (sense) and 5’-GCACCTTGAAGCGCATGAACTCCTTGATGA-3 (antisense). Reporter expression was observed in individual clones by in vitro differentiation to select for optimal expressing cell lines.

### 2.3. FACS sorting and analyses

ESCs were washed twice with cold PBS then digested using Accutase (StemCell Technologies) and centrifuged at 300g for 5 min. Cells were then resuspended in FACS staining buffer (PBS, 0.5% BSA, 2 mM EDTA, pH 7.2) and sorted for *Tbx6-EYFP* and *T-Cherry* reporter expression. FACS sorting was carried out using a FacsAria II.

For FACS analyses, cells were harvested in the same fashion as sorting, but were analyzed on a Becton-Dickinson LSRII flow cytometer. FACS sorting and analyses were carried out at the UCHC Flow Cytometry Core.

### 2.4. RNA purification and quantitative RT-PCR

RNA purification was carried out using the NucleoSpin RNA kit (Macherey-Nagel) according to manufacturer’s recommendations, including a genomic DNA digestion step. cDNA was prepared from 500 ng of RNA/sample using the ProtoScript II Reverse Transcriptase (New England BioLabs). QPCR was carried out using SybrGreen PCR Master Mix (BioRad) in an ABI 7900HT (Applied Biosystems). PCR primer sequences for gene expression analyses were designed using qPrimer Depot (Cui et al., 2007) (http://mouseprimerdepot.nci.nih.gov/), a database of optimized primers for RefSeq genes (Table 1).

### 2.5. Microscopy and imaging

Cells in culture were imaged using a Zeiss Observer Z.l inverted microscope. Fluorescence was detected using the following filter sets (Chroma Technology): HQ 500/20, HQ535/30, Q5151p, for EYFP, and HQ577/20×, HQ640/40m, Q5951p for Cherry fluorescent protein. Images were captured using an Axiocam MRc digital camera and Zen software (Zeiss).

### 2.6. Statistical analyses

Quantitative realtime PCR data are presented as mean ± standard error of the mean (SEM). Differential gene expression between groups was statistically analyzed with a t-test (CFX Manager Software 3.1; Bio- Rad Laboratories, Munich, Germany). Differences were considered statistically significant at *P* < .05 (*).

## 3. Results

### 3.1. Examination of paraxial mesoderm induction in a dual reporter mouse ESC line

**Fig. 1.**
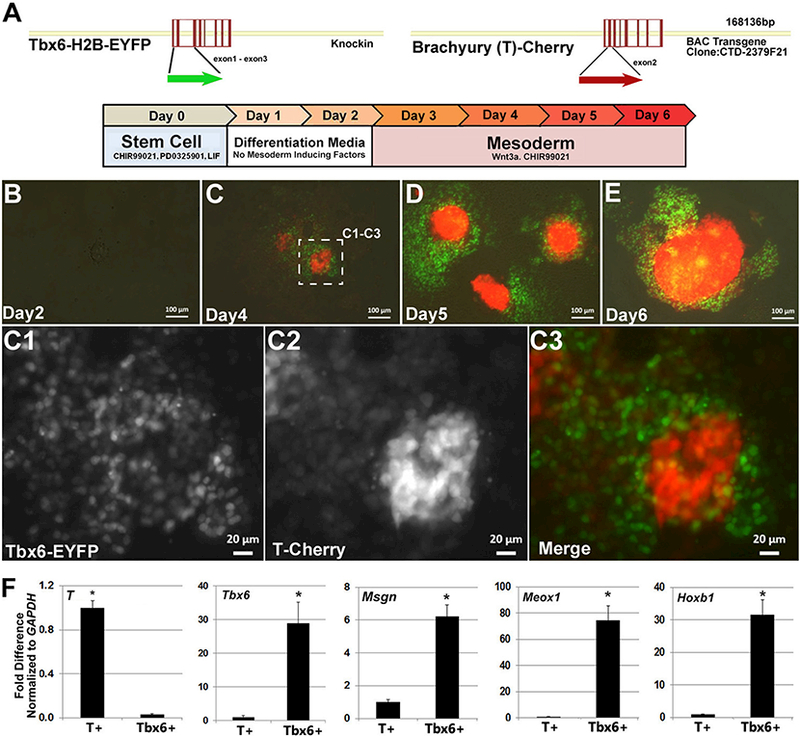
Paraxial mesoderm induction in a *Tbx6* and *Brachyury* dual reporter mouse ESC model. (A) DNA maps of reporter genes and mesoderm differentiation strategy. *Tbx6*-H2B-EYFP was targeted into the endogenous gene locus as previously described [1]. The *Brachyury-Cherry* reporter was introduced into ESCs as a BAC transgene. Established stem cells were plated for differentiation and allowed to adjust to the base differentiation media for two days, followed by four days of differentiation with mesoderm inducing factors. (B-E) Imaging of *Tbx6* (green) and *Brachyury* (red) reporter expression during ESC differentiation in réponse to Wnt3a and CHIR99021 from days 2 to 6. (C1-C3) Imaging of reporter expression at higher magnification on day 4 suggests that *Tbx6*^+^ and *Brachyury*^+^ cells are largely distinct populations. (F) Gene expression analyses on sorted cell populations confirms the fidelity of reporter expression with endogenous gene expression and enrichment of paraxial mesoderm genes in the *Tbx6*^+^ population. Data shown as mean ± SEM (*n* = 3, *p < .05, where * denotes the comparison of T^+^ to Tbx6^+^). (For interpretation of the references to colour in this figure legend, the reader is referred to the web version of this article.)

To investigate the process of paraxial mesoderm formation from mouse ESCs, we obtained a *T-box 6-H2B-EYFP (Tbx6-EYFP,* green) knock-in ESC reporter line(van den Brink et al., 2014) and introduced a *Brachyury-Cherry* BAC reporter (*T-Cherry*, red) into this cell line to generate dual reporter *T-Cherry/Tbx6-EYFP* ESCs (Fig. 1A). During embryogenesis, *Brachyury* is transiently, but broadly expressed across many different mesoderm subtypes(Wilkinson et al., 1990), while *Tbx6* is more selectively expressed in early forming paraxial mesoderm cells (Chapman et al., 1996). To verify reporter line functionality, mesoderm formation was induced by activating Wnt signaling as previously reported (Craft et al., 2013; Gadue et al., 2006; Lindsley, 2006; Tanaka et al., 2009; Umeda et al., 2012). For differentiation, mouse ESCs were seeded at low density and allowed to attach and grow for two days. After two days, canonical Wnt signaling was activated via the addition of Wnt3a (50 ng/ml) and/or CHIR99021 (3uM) over the next four days (days 2–6, Fig. 1). As anticipated, Wnt pathway activation increased the expression of both reporters over the four-day treatment period. Interestingly, the location and organization of cells expressing *T* and *Tbx6* reporters were noticeably different (Fig. 1, C-E). Strong T-Cherry reporter expression appeared at the center of the colony and over time, this group of cells increased three dimensionally in size as a spherical mass. In contrast, the majority of Tbx6^+^ cells were present around the periphery as a flatter cellular monolayer. At earlier stages of differentiation, weak T-Cherry reporter expression could also be detected in the Tbx6^+^ cell population (Fig. 1, C1-C3).

To confirm the fidelity of reporter expression, cultures were FACS sorted for Tbx6^+^ and T^+^ cells and endogenous gene expression for these respective reporters were examined (Fig. 1, D and E). T was ~33 fold higher in the T-Cherry population compared to Tbx6-EYFP cells. In contrast, Tbx6 was nearly ~30 fold higher in the Tbx6-EYFP population compared to T-Cherry cells. Given our interest in paraxial mesoderm, we also examined the expression of *Mesenchyme Homeobox 1* (*Meoxl), Hoxbl,* and *Mesogenin (Msgn1)* and found that all three gene markers were highly enriched in the Tbx6^+^ cell population (Fig. 1F). Thus, the gene expression analyses on sorted T and *Tbx6* cell populations supported the fidelity of these reporter genes.

### 3.2. Inverse agonism of retinoic acid receptors augments mesoderm induction

**Fig. 2.**
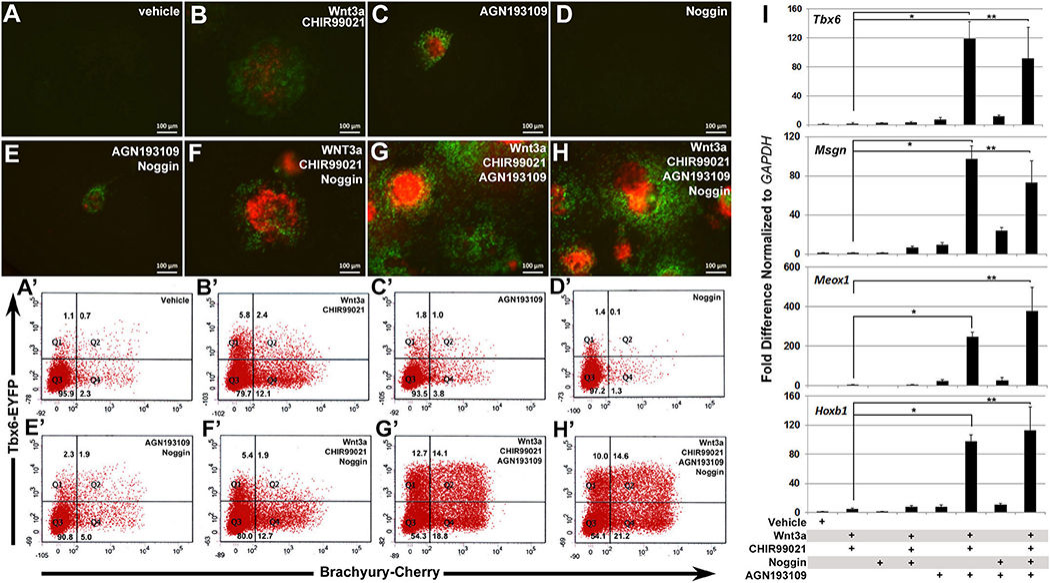
Inverse agonism of RARs augments mesoderm induction. (A-H) Representative images of reporter expression under separate and combined treatment conditions for Wnt3a/CHIR99021, AGN193109, and Noggin after 2 days of treatment (day 4 of differentiation). (A’-H’) FACS analyses of *Tbx6* and *Brachyury* reporter expressing cells after 3 days of treatment (day 5 of differentiation) under the same treatment combinations as shown in (A). RAR inhibition resulted in substantial increases in the percentage of Tbx6^+^, T^+^, and Tbx6^+^/T^+^ cells. Gates were set based on undifferentiated control stem cells. (I) Gene expression analyses on day 5 for paraxial mesoderm gene markers *Tbx6, Msgn1, Meox1,* and *Hoxb1* using indicated combinations of Wnt3a/CHIR99021, AGN193109, and Noggin. Dramatic up- regulation of all paraxial gene markers was observed when AGN193109 was added with Wnt3a/CHIR99021 with or without Noggin relative to their respective individual treatments. Data shown as mean ± SEM (n = 3, *p < .05, **p < .05, where * and ** denotes the comparison of treatments between Wnt3a, CHIR99021 to Wnt3a, CHIR99021 with AGN193109).

A large body of work has demonstrated the importance of repressing retinoic acid signaling for proper tail bud elongation (Abu-Abed et al., 2001; Fujii, 1997; Griffith and Wiley, 1991; Sakai et al., 2001; Shum et al., 1999). Additionally, repression of BMP signaling has also been shown to promote the formation of paraxial mesoderm over lateral plate mesoderm (Murtaugh et al., 1999; Tanaka et al., 2009). Therefore, we decided to test AGN193109, a pan-RAR inverse agonist and Noggin, a BMP inhibitor, for their ability to promote paraxial mesoderm formation in the presence and absence of Wnt3a/CHIR99021 treatment. Gross evaluation of *Tbx6* reporter expression (Fig. 2 A-H, green) in living cultures only 2 days after treatment revealed a noticeable benefit when AGN193109 (lμM) was combined with Wnt3a/CHIR99021 (Fig. 2, compare G and H to A-F). In contrast, there was no apparent benefit to Noggin (100 ng/ml) treatment. To more quantitatively assess *Tbx6* and *T* reporter expression, cultures were harvested and analyzed by FACS analyses three days after treatment. Consistent with our observations, FACS analyses showed that AGN193109 significantly augmented the ability of Wnt3a/CHIR99021 to stimulate mesoderm formation (Fig. 2. compare G’ and H’ to A’-F’). Relative to Wnt3a/ CHIR99021 alone, the addition of AGN193109 not only increased the T^+^ (12.2% to 18.8%) and Tbx6^+^ (5.8% to 12.7%) cell populations, but also increased the T^+^/Tbx6^+^ (2.4% to 14.1%) cell population. However, inverse agonism of RARs alone only had a marginal impact on mesoderm induction (Fig. 2C’)- FACS analyses also showed that Noggin treated cultures had no benefit with regard to promoting the early formation of paraxial mesoderm (Fig. 2, compare B’ to F’).

The synergism of AGN193109 with Wnt3a/CHIR99021 treatment was also evaluated by gene expression analyses (Fig. 21). For these studies, we examined the expression of paraxial mesoderm gene markers *Tbx6, Msgn1, Meox1,* and *Hoxb1.* Consistent with the assessment of *Tbx6* reporter expression in culture and by FACS analyses, the combined treatment of AGN193109 with Wnt3a/CHIR99021 substantially augmented the expression of all four paraxial mesoderm gene markers relative to control and individual treatments. However, the combined versus individual treatment of AGN193109 with Wnt3a/CHIR99021 did not increase *T* expression, which is not restricted to paraxial mesoderm (data not shown).

### 3.3. Epiblast state enables more efficient differentiation into paraxial mesoderm

**Fig. 3.**
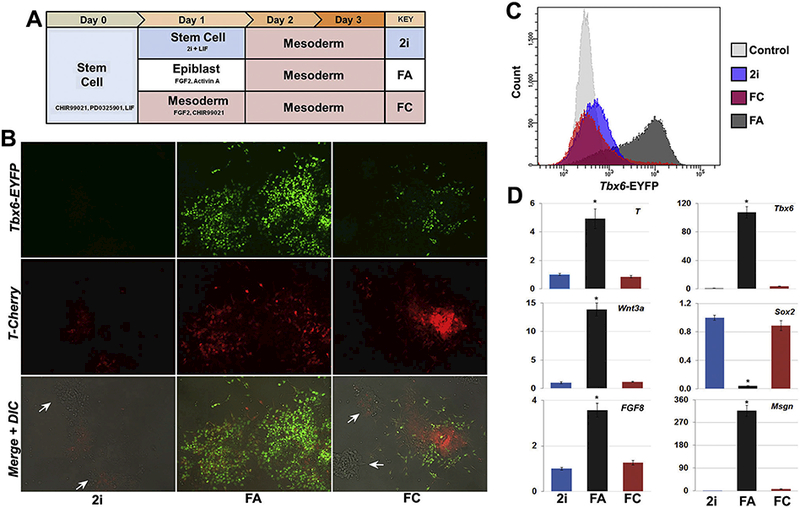
Conversion of mouse ESCs to epiblast state enhances paraxial mesoderm differentiation. (A) Schematic depiction of differentiation conditons. Note day 1 conditions for comparing delayed (2i), direct (FC), and epiblast (FA) transitions into mesoderm. (B) Representative images of reporter expression on day 3 of differentiation showing robust *Tbx6* reporter expression and spreading of *T*^+^ cells following epiblast (FA) transition. *T*^+^ cells remained clustered following direct mesoderm induction (FC), with limited Tbx6 reporter expression. The persistence of numerous mounded, reporter-negative colonies occurred with the 2i and FC transitions (white arrows), which were absent from epiblast-transitioned cultures. (C) Day 3 FACS analysis shows epiblast transition (FA) followed by two days of mesoderm differentiation generated considerably more *Tbx6*^+^ cells compared to just two (2i) or three (FC) days of mesoderm differentiation, with control stem cells shown for comparison. (D) Day 3 gene expression indicated elevated levels of early mesoderm genes T, *Wnt3a,* and *Fgf8* and substantial increases in the paraxial markers *Tbx6* and *Msgn* with FA transition compared to 2i or FC, which also retained expression *Sox2.* Data shown as mean ± SEM (n = 3, *p < .05, where * denotes the comparison of FA treatment to both 2i and FC).

While Wnt activation in conjunction with RAR inverse agonism did have a combinatorial benefit to promoting the formation of paraxial mesoderm, the overall efficiency of differentiating mouse ESCs directly into mesoderm cells was rather modest. Further, visual aspects of the differentiation process suggested to us that perhaps the mouse stem cell state was not the ideal “cell state” from which to derive mesoderm. First, adding Wnt3a and CHIR99021 earlier than day 2 did not result in faster differentiation (data not shown and Fig. 3), suggesting drifting away from the stem cell state may be required for mesoderm formation. Second, even with the delayed addition of Wnt agonists at Day 2 of culture, the response of mouse ESCs colonies was still quite variable. Some attached colonies formed spherical mounds with no reporter expression, while other colonies formed T^+^ spherical mounds (Figs. 1,2). We also noted that mouse ESC colonies that stayed flatter favored the formation of cells expressing the *Tbx6* reporter. This latter detail suggested that first converting mouse ESCs into epiblast-like cells, which unlike mouse ESCs grow in monolayer, could be more conducive to forming paraxial mesoderm.

To test this idea, we compared mouse ESCs directly differentiated into mesoderm to mouse ESCs first differentiated into epiblast-like cells by treating with FGF2 and Activin A prior to switching to mesoderm conditions (Fig. 3A). Surprisingly, these studies revealed that just a single day of differentiation towards the epiblast state followed by mesoderm induction resulted in a dramatic enhancement in the formation of Tbx6^+^ cells (Fig. 3). Imaging of Tbx6 reporter gene expression two days after mesoderm induction revealed how transitioning into the epiblast state noticeably increased the abundance of Tbx6 ^+^ cells (Fig. 3B, compare 2i or FC to FA). More quantitative metrics by FACS revealed that with one day of epiblast treatment, the percentage of Tbx6^+^ cells increased to 65–75% (Fig. 3C). Gene expression analyses showed paraxial mesoderm marker genes *Tbx6* and *Msgn* were highly expressed in mesoderm following epiblast differentiation. In contrast, mouse embryonic stem cells that were transitioned into mesoderm for two or three days had much lower levels of *Tbx6* and *Msgn,* and retained expression of *Sox2,* suggesting many cells in the culture did not undergo mesoderm differentiation. Consistent with this thinking, FACS analyses and imaging showed a prevalence of cells expressing neither the T nor Tbx6 reporters following direct differentiation from mESCs into mesoderm.

### 3.4. RAR inverse agonist treatment suggests two distinct roles exist in regulating early paraxial mesoderm formation

**Fig. 4.**
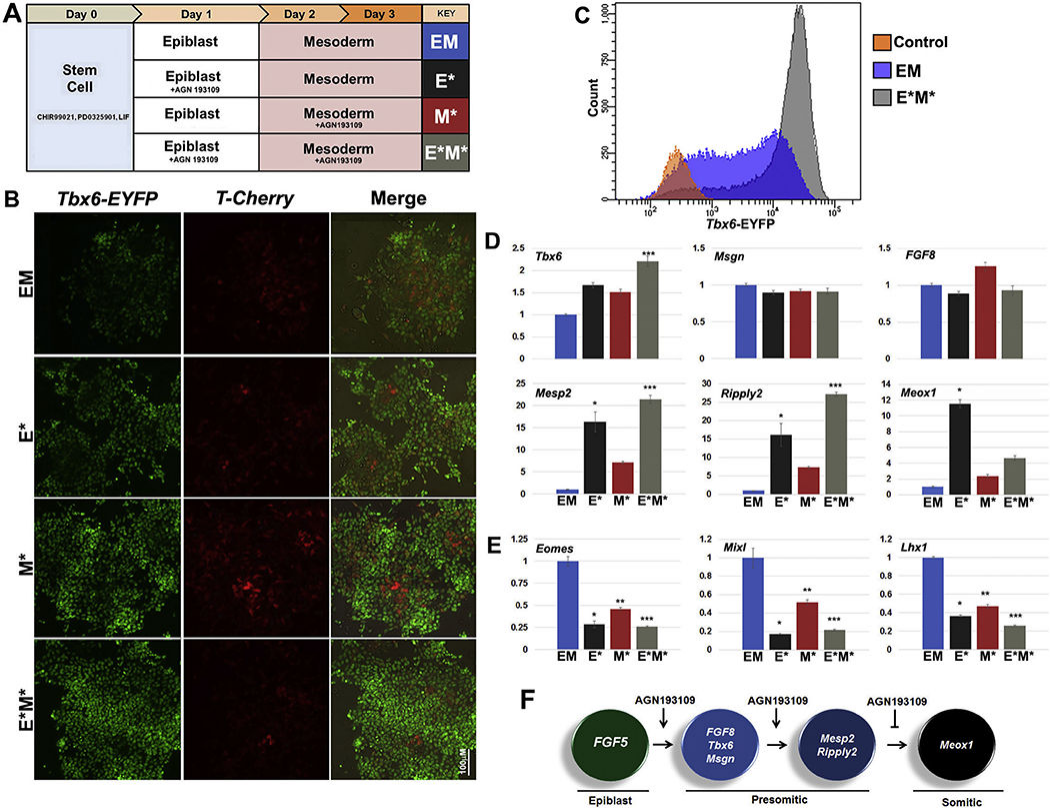
Distinct roles of RAR inverse agonist treatment in regulating paraxial mesoderm formation and maturation. (A) Schematic of differentiation conditions modifying the strategy of epiblast transition into mesoderm from Fig. 3. to assess the stage-dependent effects of AGN193109 treatment as indicated with asterisks (*). (B) Representative images of reporter expression on day 3 of differentiation showing continuous RAR inverse agonist treatment (E*M*) having the brightest level of reporter expression, confirmed by FACS analysis in (C) comparing E*M* cultures to EM and stem cell control cultures. (D) Gene expression comparing levels of early gene markers *Tbx6, Msgn,* and *FGF8* showed minimal changes between treatments. However, markers indicating a transition to somitic mesoderm, *Meox1, Mesp2,* and *Ripply2,* were remarkably higher with inverse agonist treatment, especially with treatment during epiblast stage. This benefit was also evidenced by the down- regulation of non-paraxial markers *Eomes*, *Mixl1,* and *Lhx1* in (E). (F) Model of paraxial mesoderm lineage differentiation following epiblast transition and the stage- dependent effects of AGN193109 treatment. Data shown as mean ± SI’.M *(n* = 3, ‘/) < .05, < .05, ***p < .05 where * denotes the comparison of E* to EM, ** denotes the comparison between M* to EM, and *** denotes the comparison of E*M* to EM).

With the inclusion of an epiblast differentiation step, we decided to revisit the benefit of treating cells with the RAR inverse agonist AGN193109, which promoted mesoderm differentiation (Fig. 2). However, with the addition of an epiblast differentiation step, we also wanted to determine which stage of differentiation; epiblast, mesoderm, or both, would inverse agonist treatment be more beneficial. Therefore, we compared the differentiation of mouse ESCs treated with AGN193109 at the epiblast step (E*), mesoderm step (M*), or both (E*M*) to ESCs differentiated in the absence of the inverse agonist (EM) (Fig. 4A, diagram). Visual examination of cultures showed modest increases in the extent of Tbx6 reporter expression, with continuous RAR inverse agonist treatment (E*M*) having the brightest level of reporter expression (Fig. 4B). FACS analyses substantiated these observations showing the intensity of Tbx6 reporter expression was considerably higher in E*M* treated cultures compared to EM cultures. Further, FACS analyses also showed that the continuous RAR inverse agonist treatment increased the yield of Tbx6^+^ cells to 90%, compared to 65–75% without treatment.

Gene expression analyses of paraxial gene markers also suggested that RAR inverse agonist treatment had distinct stage-dependent effects on paraxial mesoderm formation. While the examination of early gene markers such as *Tbx6, Msgn,* and *Fgf8* showed minimal changes in gene expression, gene markers associated with the transition of presomitic to somitic mesoderm *Mesp2, Ripply2, and Meox1* showed remarkable changes with inverse agonist treatment. Treatment at the epiblast stage (E*) resulted in the highest increase in *Meox1* expression at the mesoderm stage. However, continuous RAR inverse agonist treatment (E*M*) had lower levels of *Meoxl* expression compared to E*, with *Mesp2* and *Ripply2* being highly expressed.

In contrast to detecting stable or increasing expression of paraxial mesoderm marker genes, the expression of lateral plate mesoderm, intermediate mesoderm, and endoderm marker genes were either not detected or down-regulated (Fig. 4E). Lateral plate mesoderm marker genes *Tal1* and *Kdr* were undetectable. Intermediate mesoderm marker gene *Osr1* was undetectable, while *Lhx1* (Fig. 4E) was down-regulated almost 3-fold by inverse agonist treatment just at the epiblast step (E*) and 4-fold with continuous inverse agonist treatment (E*M*). Genes also important for endoderm specification *Eomes* and *Mbxl1* were down- regulated 4- and 5-fold, respectively by treatment with the inverse agonist at just the epiblast step (E*).

Taken together, this data suggests that inverse agonism of RARs at the epiblast stage promotes the future transition of epiblast cells towards the presomitic mesoderm lineage over other embryonic lineages, but continued treatment at the mesoderm step represses further maturation into somitic mesoderm (Fig. 4F). It is well established that retinoic acid signaling is involved in the maturation of presomitic unsegmented mesoderm into somitic segmented mesoderm, consistent with the lack of robust *Meox1* expression with continued treatment (Fig. 4D). Further, this likely explains why the highest expression levels and percentage of cells with *Tbx6* reporter activity were present in the E*M* treated cultures as it is known that *Tbx6* expression decreases during paraxial mesoderm maturation(Janesick et al., 2017).

### 3.5. Inverse agonism of RARs directs epiblast cells towards the paraxial mesoderm fate

**Fig. 5.**
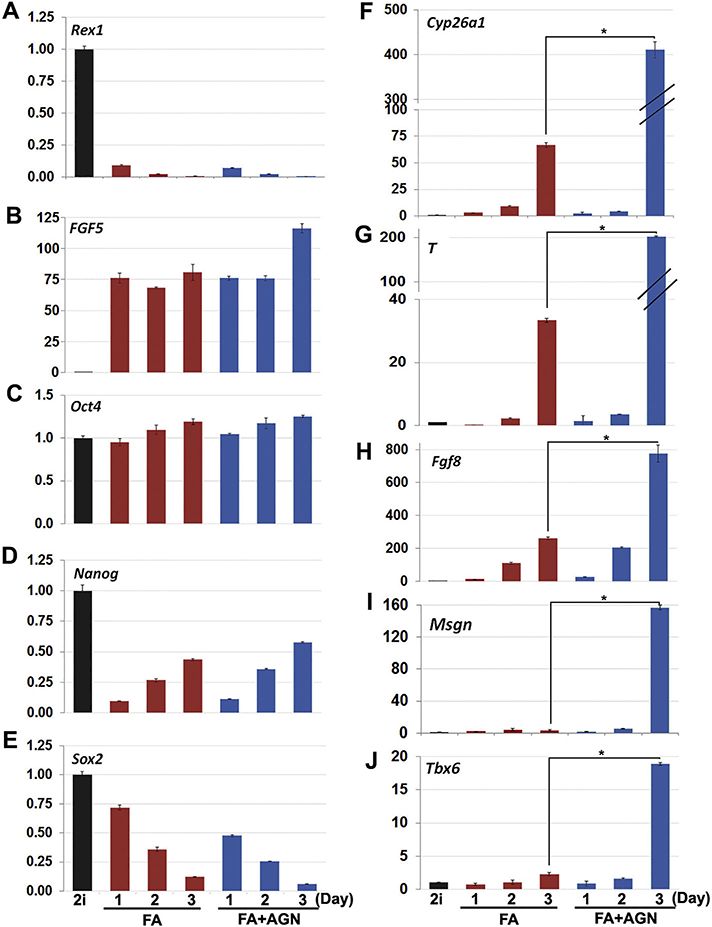
Treatment of epiblast cells with RAR inverse agonist favors a paraxial mesoderm fate. Gene expression analyses over three days of epiblast differentiation with (FA + AGN) or without (FA) inverse agonist treatment compared to stem cell (2i) control. (A,B) Rapid loss of *Rexl* expression and dramatic increase of *Fgf5* expression confirms transition from stem cell to epiblast. (C-D) *Oct4* expression remained unchanged, while a sharp drop and then gradual increase in *Nanog* expression, and a steady decrease in *Sox2* expression occurred in FA and FA + AGN conditions alike. (F-H) Caudal epiblast genes *Cyp26al, T,* and *Fgf8* were strongly increased over three days of epiblast differentiation, while including AGN193109 treatment showed even greater enhancement of expression levels. (I,J) Inverse agonist treatment also led to substantial increases in paraxial mesoderm markers *Msgn* and *Tbx6* by day 3 of epiblast differentiation not seen in the FA treatment, suggesting AGN193109 promotes epiblast differentiation into the paraxial mesoderm lineage. Data shown as mean ± SEM (n = 3, *p < .05, where * denotes the day 3 comparison between FA to FA with AGN193109).

With evidence that treatment with AGN193109 at the epiblast step of differentiation can influence the future differentiation of epiblast cells into paraxial mesoderm, we decided to examine the effect of RAR inverse agonist treatment on gene expression during epiblast differentiation. However, in contrast to the prior experiment where epiblast differentiation lasted just a single day, we decided to carry out epiblast differentiation for three days in the presence and absence of inverse agonist treatment (Fig. 5). The conversion from the stem cell state to the epiblast state was detected by the rapid down regulation of the inner cell mass marker gene *Rex1* (Fig. 5A) and the dramatic increase in the epiblast marker gene *Fgf5* (Fig. 5B). The expression of pluripotency marker gene *Oct4* remained unchanged throughout the three-day period, while *Nanog* expression abruptly dropped, but then gradually increased (Fig. 5C,D). The pluripotency marker gene *Sox2* gradually decreased over the three-day differentiation period with slightly lower expression levels with inverse agonist treatment. Collectively, the examination of these gene markers did not reveal any dramatic changes in gene expression in the addition of AGN193109 treatment.

However, examination of caudal epiblast genes *Cyp26a1, T,* and *Fgf8* and early markers of paraxial mesoderm *Msgn* and *Tbx6* did reveal that inverse agonist treatment was somehow promoting meaningful changes in epiblast gene expression (Fig. 5F-J). By day three of epiblast differentiation in the presence of inverse agonist treatment, *Cyp26a1*and *T* were 6-fold higher (Fig. 5F,G), *Fgf8* was 3-fold higher (Fig. 5H), and *Msgn* and *Tbx6* were 45- and 9-fold higher, respectively, compared to epiblast conditions alone (Fig. 5I,J). This delayed yet pronounced increase in a subset of caudal epiblast marker genes that are largely responsible for driving axial growth suggests that inverse agonism of RARs in epiblast cells can promote their direction of differentiation towards the paraxial mesoderm cell lineage.

## 4. Discussion

In this study, we showed that the differentiation of paraxial mesoderm directly from the mouse ESC “naïve” state is considerably less efficient than differentiation from the epiblast state. This work also showed how the use of AGN193109, a pan-RAR inverse agonist, when added at the epiblast and mesoderm steps of differentiation promoted the formation of paraxial mesoderm over other mesoderm and endoderm cell lineages. Further, continued treatment of AGN193109 during mesoderm differentiation repressed the maturation of pre- somitic mesoderm into somitic mesoderm. This two-step, three-day differentiation protocol resulted in an extremely high yield of Tbx6^+^ paraxial mesoderm cells (90%) from mouse ESCs. Taken together, this work introduces a novel and efficient approach to generate paraxial mesoderm from mouse ESCs and suggests a very early retinoic acid independent role exists for RARs in epiblast cells and early mesoderm progenitor cells that promotes their differentiation into the paraxial mesoderm lineage.

### 4.1. Epiblast state is a better staging ground for generating paraxial mesoderm

While our studies and work by others (Jackson et al., 2010; Lindsley, 2006; Tanaka et al., 2009; Thomson et al., 2011) have shown that mesoderm differentiation directly from the mouse ESC naive state is possible, we also demonstrate how paraxial mesoderm formation initiated after an epiblast transition works with greater efficiency. We speculate that the likely reason for this relates to the conflicting roles of p-Catenin as a factor that promotes both stem cell pluripotency and mesoderm differentiation (Cunningham et al., 2016; Hoffman et al.,2013; Kim et al., 2013; Lindsley, 2006; Wray et al., 2011; Zhang et al., 2013). CHIR99021, the GSK3 inhibitor that results in β-Catenin stabilization is one of two sternness molecules in 2i stem cell maintenance media, but also is a widely used molecule for mesoderm differentiation (Buehr et al., 2008; Gouti et al., 2014; Thomson et al., 2011; Ying et al., 2008). Indeed, at the DNA level, β-Catenin has been shown to occupy regulatory regions on both sternness and mesoderm inducing genes in mouse ESCs (Zhang et al., 2013). Further, in naïve cells β-Catenin represses TCF3 function to maintain the naïve state, while TCF3 is important for the transition from naïve to the primed epiblast state (Hoffman et al., 2013; Wray et al., 2011).

With this in mind, we and others (Jackson et al., 2010; Thomson et al., 2011) have noted that mouse ESCs do not immediately respond to mesoderm inducing factors, as the removal of pluripotency conditions for up to 48 h is often necessary to ensure a higher percentage of cells exit the stem cell state and effectively respond to mesoderm inducing factors. In contrast, we show that naïve mouse ESCs can rapidly and uniformly respond to epiblast differentiation conditions, indicating that FGF and Activin signaling pathways do not provide conflicting cues that would promote the naïve state, but in fact counter naïve sternness regulators to allow uniform differentiation into the epiblast state. While pluripotency genes are still expressed at the epiblast state, how they are regulated has markedly changed and is independent of p-catenin regulation (Chen et al., 2008; Greber et al., 2010; Hoffman et al., 2013; Tesar et al., 2007). Thus, the stabilization of p-catenin via treatment of CHIR99021 following epiblast conversion no longer provides conflicting signals to promote sternness and mesoderm, thereby allow efficient mesoderm differentiation to be initiated.

### 4.2. Inverse agonism of RARs steers epiblast cells towards a caudal fate

In this study we show how the addition of an RAR inverse agonist, AGN193109, promoted the expression of caudal epiblast marker genes as well as early paraxial mesoderm marker genes (Fig. 4). However, the mechanism(s) behind this effect remain unclear. Work by others has shown that when inverse agonists bind to RARs, a change in protein structure occurs that stabilizes the association of RARs with transcriptional co-repressors NCoR and SMRT (Bourguet et al., 2010; Bourguet et al., 2000; Freedman, 1999; Germain et al., 2009; Glass and Rosenfeld, 2000). In contrast, when RA binds to RARs they generally associate with transcriptional co-activators NCoA. With that in mind, in vivo studies have shown that RARs are expressed in the epiblast, which is devoid of RA signaling based on RARE-LacZ reporter characterization (Uehara et al., 2009). Thus, it is likely that RARs function in the epiblast in a RA independent manner, but their exact role in the epiblast remains unclear. The work presented here suggests that RARs may function in the absence of RA to promote caudal epiblast formation and paraxial mesoderm formation.

It is generally accepted that treatment with RAR inverse agonists is thought to mimic the unliganded conformation and function of RARs; as in the absence of RA, RARs have been shown to associate with corepressors NcoA and SMRT (Bourguet et al., 2000; Freedman, 1999; Glass and Rosenfeld, 2000; Weston et al., 2003). Therefore, it is conceivable that differentiation performed in the absence of vitamin A, the precursor to RA, should mimic the effects of inverse agonist treatment. In support of this thinking, mouse ESCs deficient of *Aldhla2,* which encodes for an enzyme necessary for the synthesis of RA, showed substantial up-regulation of early paraxial mesoderm gene markers *Tbx6* and *Msgn* (Gouti et al., 2017). However, we performed our differentiation studies in vitamin A deficient media and the addition of inverse agonist still had noticeable benefits to paraxial mesoderm formation. In fact, the up-regulation of *Tbx6* and *Msgn* during prolonged epiblast differentiation appeared to be highly dependent on the addition of AGN193109 (Fig. 5). With that said, mouse ESCs are commonly maintained in serum free media containing vitamin A, and retinol derivatives stored inside cells (Metzler and Sandell, 2016)may persist over the initial days of differentiation, which may impact early cell fate decisions. Also, work by others suggests vitamin A is a valuable component to the sternness and growth of mouse ESCs. RA-independent and possibly RA-dependent roles for vitamin A are important for maintaining rates of mouse ESC proliferation and pluripotency (Chen et al., 2007; Chen and Khillan, 2010; Chen and Khillan, 2008; Khillan, 2014; Tagliaferri et al., 2016; Yang et al., 2015). Therefore, maintaining mouse ESCs in the absence of vitamin A is not likely a viable option, but could interfere with efficiency of subsequent differentiation steps as our studies possibly suggest. Thus, the application of RAR inverse agonists to rapidly outcompete any effects from low levels of RA to promote paraxial mesoderm differentiation is a simple solution.

### 4.3. RAR inverse agonism represses paraxial mesoderm maturation

While RAR inverse agonism at the epiblast step of differentiation promoted paraxial mesoderm induction, our studies also indicated that continuous treatment during the mesoderm differentiation step delayed further maturation. Continuous inverse agonist treatment resulted in reduced *Meox1* expression, while at the same time generated the highest percentage of Tbx6^+^ cells (90%). Consistent with our outcomes, past embryonic studies have shown that severe axial truncation occurs when excessive levels of retinoic acid signaling occurs (Griffith and Wiley, 1991). Excessive levels of RA overcome the threshold of Cyp26al degradation and bind RAR0γ resulting in truncation (Lohnes et al., 1993). In contrast to RA treatment and similar to our outcomes, in vivo studies in Xenopus have shown that the addition of pan-RAR and RARγ specific inverse agonists delayed the maturation of unsegmented paraxial mesoderm to maintain a caudal progenitor pool (Janesick et al., 2014). While the benefit of continuous inverse agonist treatment at the mesoderm step could be argued, we believe it is likely that delaying paraxial mesoderm maturation during embryonic stem cell differentiation will maintain better synchrony among cells in culture, which will provide a more uniform cellular response for subsequent differentiation steps.

In these studies, we highlight the benefit of epiblast differentiation and RAR inverse agonism as an approach to increase paraxial mesoderm formation from mouse ESCs. By enhancing the guidance of ESCs into paraxial mesoderm, downstream efforts to generate different skeletal cell types will be improved.

## Figures and Tables

**Table 1.** QPCR primers.

Gene symbol	Sense (forward)	Antisense (reverse)	Species
*Gapdh*	5’-CGTCCCGTAGACAAAATGGT-3’	5’TTGATGGCAACAATCTCCAC3’	Mouse
*Tbx6*	5’-TGACAGCCTACCAGAACCCT-3’	5’-CCCGAAGTTTCCTCTTCACA-3’	Mouse
*Msgn1*	5’-AACCTGGGTGAGACCTTCCT-3’	5’-TCCGCATCCTGAGTTTCTCT-3’	Mouse
*Hoxb1*	5’-TTCGACTGGATGAAGGTCAA-3’	5’-GGTGAAGTTTGTGCGGAGAC-3’	Mouse
*Meox1*	5’-GCCAATGAGACGGAGAAGAA-3’	5’-TTGGTGAAGGCTGTCCTCTC-3’	Mouse
*Sox2*	5’-ACAAGAGAATTGGGAGGGGT-3’	5’-AAGCGTTAATTTGGATGGGA-3’	Mouse
*T*	5’GTCTAGCCTCGGAGTGCCT3’	5’-CCATTGCTCACAGACCAGAG-3’	Mouse
*Fgf8*	5’-GCTCATTGTGGAGACCGATA-3’	5’-AATACGCAGTCCTTGCCTTT-3’	Mouse
*Wnt3a*	5’-ACTACGTGGAGATCATGCCC-3’	5’-GGTGGCTTTGTCCAGAACAG-3’	Mouse
*Mesp2*	5’-TGGACACAATCCACTGAACC-3’	5’-GGCTGTAGTCTCTGGCATGA-3’	Mouse
*Ripply2*	5’-ATGGATACCACCGAGAGCGCCGAGA-3’	5’-GGTACCCGGGCTGCGCGGAC-3’	Mouse
*Mixl1*	5’CGACAGACCATGTACCCAGA3’	5’-CCTTGAGGATAAGGGCTGAA-3’	Mouse
*Eomes*	5’GGCCTACCAAAACACGGATA3’	5’-GACCTCCAGGGACAATCTGA-3’	Mouse
*Lhx1*	5’TGTAAATGCAACCTGACCGA-3’	5’-AACCAGATCGCTTGGAGAGA-3‘	Mouse
*FgfS*	5’GCTGTGTCTCAGGGGATTGT-3’	5’-ACAGTCATCCGTAAATTTGGC-3’	Mouse
*Nanog*	5’-AAGTACCTCAGCCTCCAGCA-3’	5’-GTGCTGAGCCCTTCTGAATC-3’	Mouse
*Oct4*	5’-AGAGGGAACCTCCTCTGAGC-3’	5’-TTCTAGCTCCTTCTGCAGGG-3’	Mouse
*Cyp26a1*	5’-GCAGGAAATACGGCTTCATC-3’	5’-ATCACCTTCTTTCGCTGCTT-3’	Mouse
*Rex1*	5’TGAAAGTGAGATTAGCCCCGAG3’	5’-GTCCCATCCCCTTCAATAGCAC-3’	Mouse
